# The Value of Admission Serological Indicators for Predicting 28-Day Mortality in Intensive Care Patients With Acute Heart Failure: Construction and Validation of a Nomogram

**DOI:** 10.3389/fcvm.2021.741351

**Published:** 2021-12-03

**Authors:** Xiaoyuan Wei, Yu Min, Jiangchuan Yu, Qianli Wang, Han Wang, Shuang Li, Li Su

**Affiliations:** ^1^Department of Cardiology, The Second Affiliated Hospital, Chongqing Medical University, Chongqing, China; ^2^Department of Breast and Thyroid Surgery, The Second Affiliated Hospital of Chongqing Medical University, Chongqing, China

**Keywords:** acute heart failure, mortality, serological examination, nomogram, MIMIC III, intensive care unit

## Abstract

**Background:** Acute heart failure (AHF) is a severe clinical syndrome characterized as rapid onset or worsening of symptoms of chronic heart failure (CHF). Risk stratification for patients with AHF in the intensive care unit (ICU) may help clinicians to predict the 28-day mortality risk in this subpopulation and further raise the quality of care.

**Methods:** We retrospectively reviewed and analyzed the demographic characteristics and serological indicators of patients with AHF in the Medical Information Mart for Intensive Care III (MIMIC III) (version 1.4) between June 2001 and October 2012 and our medical center between January 2019 and April 2021. The chi-squared test and the Fisher's exact test were used for comparison of qualitative variables among the AHF death group and non-death group. The clinical variables were selected by using the least absolute shrinkage and selection operator (LASSO) regression. A clinical nomogram for predicting the 28-day mortality was constructed based on the multivariate Cox proportional hazard regression analysis and further validated by the internal and external cohorts.

**Results:** Age > 65 years [hazard ratio (HR) = 2.47], the high Sequential Organ Failure Assessment (SOFA) score (≥3 and ≤8, HR = 2.21; ≥9 and ≤20, HR = 3.29), lactic acid (Lac) (>2 mmol/l, HR = 1.40), bicarbonate (${\text{HCO}}_{3}^{-}$) (>28 mmol/l, HR = 1.59), blood urea nitrogen (BUN) (>21 mg/dl, HR = 1.75), albumin (<3.5 g/dl, HR = 2.02), troponin T (TnT) (>0.04 ng/ml, HR = 4.02), and creatine kinase-MB (CK-MB) (>5 ng/ml, HR = 1.64) were the independent risk factors for predicting 28-day mortality of intensive care patients with AHF (*p* < 0.05). The novel nomogram was developed and validated with a promising C-index of 0.814 (95% CI: 0.754–0.882), 0.820 (95% CI: 0.721–0.897), and 0.828 (95% CI: 0.743–0.917), respectively.

**Conclusion:** This study provides a new insight in early predicting the risk of 28-day mortality in intensive care patients with AHF. The age, the SOFA score, and serum TnT level are the leading three predictors in evaluating the short-term outcome of intensive care patients with AHF. Based on the nomogram, clinicians could better stratify patients with AHF at high risk and make adequate treatment plans.

## Introduction

Heart failure (HF) is one of the most frequent cardiovascular-related diseases in modern society, which influences the growing number of populations around the world (1–3). Moreover, HF is fundamentally recognized as one of the leading causes of hospitalization among patients aged > 65 years of age in the United States. For hospitalizations with primary HF, the estimated average cost was nearly $11,552 in 2014, totaling an estimated $11 billion (1, 3, 4). In contrast to the great improvements in the treatment of chronic heart failure (CHF), acute heart failure (AHF) is still associated with a worse prognosis, with 90-day readmission rates and 1 year mortality reaching 10–30% in the United States (2, 5). In the rest of the world, the mortality in Africa and India was determined with the highest rate of 34 and 23%, respectively, about mean mortality in Southeast Asia (15%) and the lowest mortality in China (7%), South America (9%), and the Middle East (9%) (6).

Thus, the risk stratification of AHF based on different clinical characteristics and biomarkers has been proposed. The ideal risk stratification system would identify a subpopulation of patients with similar pathophysiology and clinical presentation at admission, so that treatment may be tailored for each patient. During the past years, researchers from the different regions have made some attempts to discover the prognostic factors in predicting the short-term (28–30-days) or long-term (1–5 years) mortality and readmission in patients with AHF (7–17). Notably, reviewing the recently published literature, elderly age, multimorbidity, high blood urea nitrogen (BUN), serum creatinine, and hypoalbuminemia were significantly associated with the increased risk of AHF-related mortality (2, 3). Additionally, HIV positive was also identified as a meaningful evaluation indicator for patients with AHF from Africa (18). On the other hand, readmission (10) and lower health literacy (19) were also identified as indicators of long-term mortality after discharge. Compelling evidence demonstrated that delayed treatment delivery is associated with poor outcomes in AHF (2). Thus, following the concept of “time-to-treatment,” early identifying patients with AHF at high risk of mortality at initial admission to the intensive care unit (ICU) could significantly help clinicians to achieve a timely diagnosis and individualized treatment modality.

In this study, we aim to determine the prognostic factors for predicting the 28-day mortality in intensive care patients with AHF at admission. Based on the admission serological indicators, we further aim to establish an individualized nomogram for routine clinical use.

## Materials and Methods

### Data Source

The data of this study was from one public database and our medical center. Specifically, the training data with respect to clinical characteristics of patients with AHF were obtained from the Medical Information Mart for Intensive Care III (MIMIC III) (version 1.4), derived from a large, freely accessible critical care database comprising deidentified health records (58,976 hospitalization records) of 46,520 patients who were admitted to the ICU of Beth Israel Deaconess Medical Center between June 2001 and October 2012 (https://mimic.physionet.org/). The cases of the internal validating cohort were produced by 1,000 resampling bootstrap analyses from the training data. Besides, the external validation cohort was collected from hospitalizations of the Second Affiliated Hospital of Chongqing Medical University during January 2019 and April 2021.

### Ethics Approval

The protocol for this study was approved by Chongqing Medical University. Ethical approval was waived by the local Ethics Committee of the Chongqing Medical University in view of the retrospective nature of this study and all the procedures being performed were part of the routine care.

### Patient Selection

**Figure 1:**
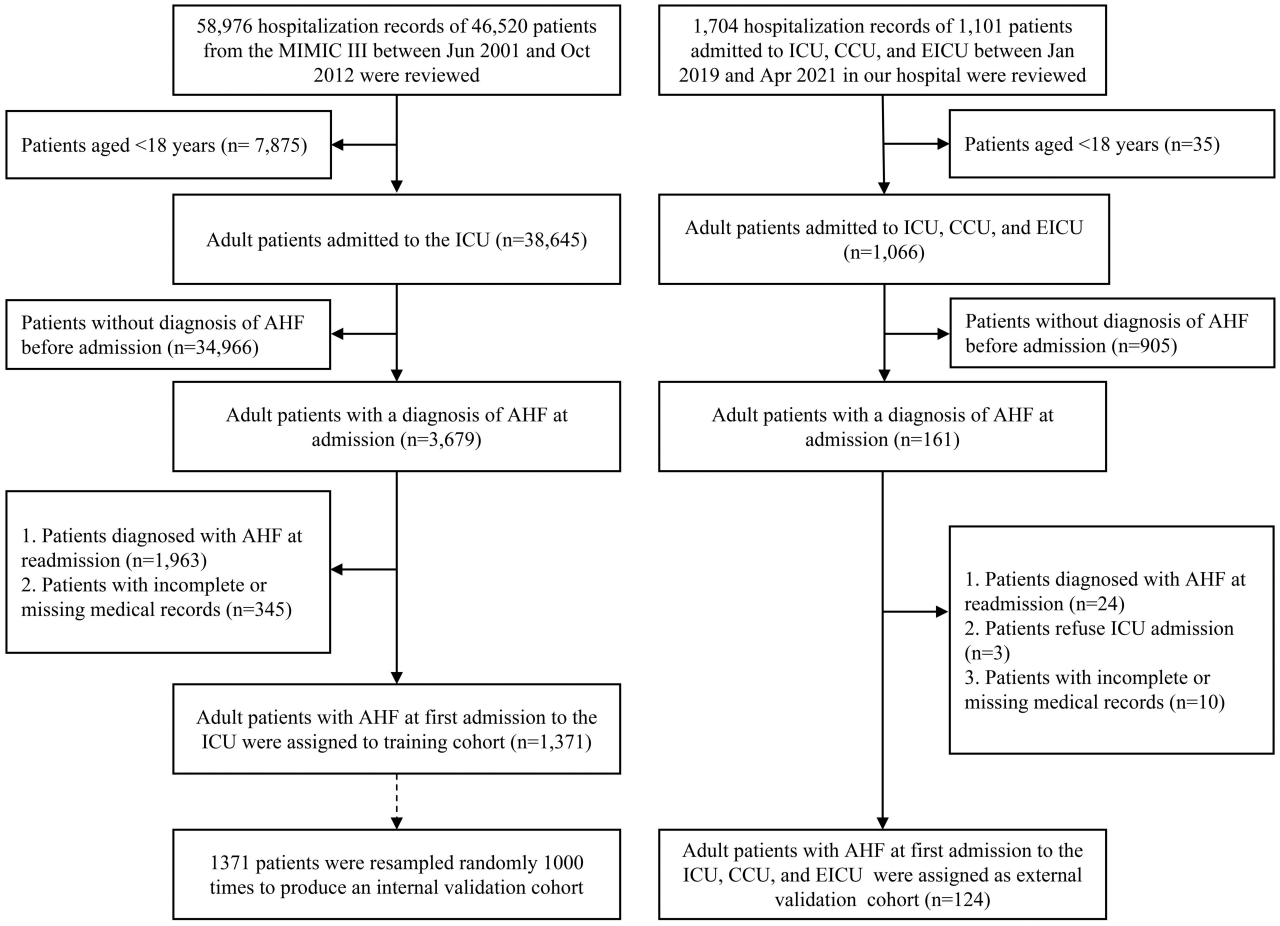
The patient selection process for this study. AHF, acute heart failure; ICU, intensive care unit; CCU, coronary care unit; EICU, emergency ICU.

In the MIMIC III program, we retrospectively screened patients diagnosed with AHF before admitting them to the ICU. In our medical record system, we identified patients with AHF at first admission between January 2019 and April 2021 in the ICU, emergency ICU (EICU), and coronary care unit (CCU) (Figure 1).

### Diagnosis of AHF

Acute heart failure is often accompanied by other morbidities and the diagnosis of AHF is frequently made clinically based on history and clinical signs (1, 3, 6). On one hand, in the MIMIC III database, the diagnosis of AHF was following the codes: icd9_code: 42821, 42823, 42831, 42833, 42841, and 42843. On the other hand, in our department, the diagnosis of AHF was based on the detailed medical history of heart disease or multimorbidity combined with some of the symptom and clinical signs including dyspnea on exertion, lower extremity edema, orthopnea, paroxysmal nocturnal dyspnea, reduced exercise tolerance, jugular vein distension, pulmonary rale, cold and clammy skin, and the presence of a third heart sound. Besides, suspicious patients with AHF are further evaluated by examinations including but not limited to natriuretic peptides, ECGs, and echocardiography.

### Criteria to Intensive Care

The common criteria for admission to an ICU or a CCU include: (i) hemodynamic instability (heart rate < 40 beats/min or >130 beats/min); (ii) systolic blood pressure < 90 mm Hg or evidence of hypoperfusion and respiratory distress (respiratory rate > 25 breaths/min, peripheral oxygen saturation < 90% despite supplemental oxygen); and (iii) use of accessory muscles for breathing or need for mechanical ventilatory support (20).

### Variable Evaluation

The proportional hazards assumption was assessed by plotting Schoenfeld residuals vs. time and examining their correlation (Supplementary Figure 1). The continuous variables in this study did not satisfy the proportional hazards assumption (*p* < 0.05). Thus, according to the normal reference values of indicators in the MIMIC III database, all the continuous variables were analyzed as categorized.

#### Clinical Baseline Information

The sex (female and male), age (>18 and ≤65 years and >65 years), race (white, black, and other), hypertension (yes and no), chronic obstructive pulmonary disease (COPD) (yes and no), diabetes (yes and no), acute myocardial infarction (AMI) (yes and no), and body mass index (BMI) (normal: ≥18 and <24 kg/m^2^) were reviewed.

#### Severity Scores

The Sequential Organ Failure Assessment (SOFA) score was analyzed individually according to the severity of system impairment (including neurologic, renal, cardiovascular, respiratory, coagulation, and hepatic) and each organ system got a score that ranges between 0 and 4. They were divided into three groups: total scores: ≥0 and <2, ≥3 and ≤8, and ≥9 and ≤20.

The Glasgow Coma Scale (GCS) score was calculated according to the documented motor, verbal, and eye responses at the admission medical records. We divided into three groups: total score: ≥13 and ≤15, ≥9 and ≤12, and ≥4 and ≤8.

#### Serological Indicators

The white blood cell (WBC) (normal: ≥4 and ≤10 k/μL), hemoglobin (Hb) (normal: male > 120 g/l, female > 110 g/l), platelet (PLT) (normal: ≥100 and ≤3,001 k/μl), sodium (Na) (normal: 135–145 mmol/l), potassium (K) (normal: ≥3.5 and ≤5.5 mmol/l), chlorine (Cl) (normal: ≥96 and ≤106 mmol/l), total calcium (tCa) (normal: ≥9 and ≤11 mg/dl), base excess (BE) (normal: ≥-3 and ≤3 mmol/l), bicarbonate (${\text{HCO}}_{3}^{-}$) (normal: ≥22 and ≤28 mmol/l), anion gap (AG) (normal: ≥8 and ≤16 mmol/l), lactic acid (Lac) (normal: ≤2 mmol/l), blood urea nitrogen (BUN) (normal: ≤21 mmol/l), serum creatinine (Scr) (normal: male: <1.5 mg/dl, female: <1.0 mg/dl), albumin (ALB) (normal: ≥3.5 g/dl), total bilirubin (Tbil) (normal: ≤1.2 mg/dl), aspartate aminotransferase (AST) (normal: ≤35 U/l), alanine aminotransferase (ALT) (normal: ≤40 U/l), troponin T (TnT) (normal: ≤0.04 ng/ml), and creatine kinase-MB (CK-MB) (normal: ≤5 ng/ml) were screened out for constructing the database of this study (Table 1). The measurement unit for each variable derived from the MIMIC III database and our medical center was unified (18 × 1 mg/dl = 1 mmol/l; 1 g/dl = 10 g/l; 1 k/μl = 1 × 10^9^/L). Note: the tCa was corrected by the formula = measured total calcium (mg/dl) + 0.8 [4.0 – serum albumin (g/dl)].

**Table 1:** The demographic characteristics of patients with acute heart failure (AHF) during the first intensive care unit admission.

**Variables**	**Subgroup**	**No. (%) of patients**		
		**Training cohort** **(*n =* 1,371)**	**Internal cohort** **(*n =* 685)**	**External cohort** **(*n =* 124)**
Sex	Male	776 (56.6)	396 (57.8)	68 (54.8)
	Female	595 (43.3)	289 (42.2)	56 (45.1)
Age (years)	≤65	340 (24.7)	187 (27.3)	34 (27.4)
	>65	1,031 (75.3)	498 (72.7)	90 (72.6)
Race	White	1,019 (74.3)	516 (75.3)	/
	Black	128 (9.3)	53 (7.7)	/
	Other	224 (16.3)	116 (17.0)	124 (100.0)
Hypertension	No	827 (60.3)	423 (61.8)	64 (51.6)
	Yes	544 (39.7)	262 (38.2)	60 (48.4)
COPD	No	1,300 (94.8)	655 (95.6)	104 (83.9)
	Yes	71 (5.2)	30 (4.4)	20 (16.1)
Diabetes	No	807 (58.8)	405 (59.1)	83 (66.9)
	Yes	564 (41.2)	280 (40.9)	41 (33.1)
AMI	No	1,306 (95.2)	655 (95.6)	73 (58.9)
	Yes	65 (4.7)	30 (4.4)	51 (41.1)
SOFA (score)	≥0 and <2	305 (22.2)	162 (23.7)	21 (16.9)
	≥3 and ≤8	869 (63.4)	444 (64.8)	78 (62.9)
	≥9 and ≤20	197 (14.4)	79 (11.5)	25 (20.16)
GCS (score)	≥13 and ≤15	1,186 (86.5)	594 (86.7)	102 (82.2)
	≥9 and ≤12	106 (7.7)	57 (8.3)	18 (14.5)
	≥4 and ≤8	79 (5.8)	34 (5.0)	4 (3.2)
WBC (k/uL)	<4	32 (2.3)	17 (2.4)	5 (4.0)
	≥4 and ≤10	582 (42.4)	286 (41.8)	62 (50.0)
	>10	757 (55.2)	382 (55.8)	57 (46.0)
Hb (g/L)	Normal	424 (30.9)	205 (29.9)	50 (40.3)
	Low	947 (69.1)	480 (70.1)	74 (59.7)
PLT (k/uL)	<100	98 (7.1)	19 (2.8)	10 (8.1)
	≥100 and ≤300	1,005 (73.3)	498 (72.7)	96 (77.4)
	>300	268 (19.5)	348 (50.8)	18 (14.5)
Na (mmol/L)	Normal	1,074 (78.3)	551 (80.4)	72 (58.1)
	Abnormal	297 (21.7)	134 (19.6)	52 (41.9)
K (mmol/L)	<3.5	157 (11.5)	69 (10.1)	15 (12.1)
	≥3.5 and ≤5.5	1,125 (82.0)	567 (82.8)	102 (82.3)
	>5.5	89 (6.5)	49 (7.1)	7 (5.6)
Cl (mmol/L)	<96	146 (10.6)	80 (11.7)	32 (25.8)
	≥96 and ≤106	824 (60.1)	434 (63.4)	79 (63.7)
	>106	401 (29.2)	171 (24.9)	13 (10.5)
tCa (mg/dl)	Normal/Hypercalcemia	282 (20.6)	145 (21.2)	61 (49.2)
	Hypocalcemia	1,089 (79.4)	540 (78.8)	63 (50.8)
^adjust^tCa (mg/dl)	Normal/Hypercalcemia	605 (44.1)	357 (52.1)	82 (66.1)
	Hypocalcemia	766 (55.9)	328 (47.9)	42 (33.9)
BE (mmol/L)	<-3	259 (18.9)	118 (17.2)	41 (33.1)
	≥-3 and ≤3	877 (64.0)	429 (62.6)	68 (54.8)
	>3	235 (17.1)	138 (20.1)	15 (12.1)
${\text{HCO}}_{3}^{-}$ (mmol/L)	<22	418 (30.5)	183 (26.7)	44 (35.5)
	≥22 and ≤28	697 (50.8)	369 (53.9)	63 (50.8)
	>28	256 (18.7)	133 (19.4)	17 (13.7)
AG (mmol/L)	≥8 and ≤16	967 (70.5)	479 (69.9)	89 (71.8)
	>16	404 (29.5)	206 (30.1)	35 (28.2)
Lac (mmol/L)	≤2	1,030 (75.1)	526 (76.8)	90 (72.6)
	>2	341 (24.9)	159 (23.2)	34 (27.4)
BUN (mg/dl)	≤21	338 (24.7)	181 (26.4)	45 (36.3)
	>21	1,033 (75.3)	504 (73.6)	79 (63.7)
Scr (mg/dl)	Normal	684 (49.9)	357 (52.1)	59 (47.6)
	High	687 (50.1)	328 (47.9)	65 (52.4)
ALB (g/dl)	<3.5	1,112 (81.1)	567 (82.8)	48 (38.7)
	≥3.5	259 (18.9)	118 (17.2)	76 (61.3)
TBil (mg/dl)	≤1.2	1,074 (78.3)	538 (78.5)	104 (83.9)
	>1.2	297 (21.7)	147 (21.5)	20 (16.1)
ALT (U/L)	≤40	939 (68.5)	476 (69.5)	90 (72.6)
	>40	432 (31.5)	209 (30.5)	34 (27.4)
AST (U/L)	≤35	690 (50.3)	343 (50.1)	72 (58.1)
	>35	681 (49.7)	342 (49.9)	52 (41.9)
TnT (ng/ml)	≤0.04	691 (50.4)	335 (48.9)	75 (60.5)
	>0.04	680 (49.6)	350 (51.1)	49 (39.5)
CKMB (ng/ml)	≤5	792 (57.8)	386 (56.4)	70 (56.5)
	>5	579 (42.2)	299 (43.6)	54 (43.5)
BMI (kg/m^2^)	<24	153 (11.2)	69 (10.1)	98 (79.0)
	≥24	602 (43.9)	285 (41.6)	26 (21.0)
	NM	616 (44.9)	331 (48.3)	/
Death	No	1,145 (83.5)	575 (83.9)	94 (75.8)
	Yes	226 (16.5)	110 (16.1)	30 (24.2)

*COPD, chronic obstructive pulmonary disease; AMI, acute myocardial infarction; SOFA, Sequential Organ Failure Assessment; GCS, Glasgow Coma Scale; WBC, white blood cell; Hb, hemoglobin (normal: male > 120 g/l and female > 110 g/l); PLT, platelet; Na, sodium (normal: 135–145 mmol/l); K potassium; Cl, chlorine; tCa, total calcium (normal: ≥9 and ≤11 mg/dl); ${\text{HCO}}_{3}^{-}$, bicarbonate; BE, base excess; AG, anion gap; Lac, lactic acid; BUN, blood urea nitrogen; Scr, serum creatinine (normal: male: <1.5 mg/dl, female: <1.0 mg/dl), ALB; albumin; Tbil, total bilirubin; ALT, aspartate aminotransferase; AST, alanine aminotransferase; TnT, troponin T; CK-MB, creatine kinase-MB; BMI, body mass index; NM, not mentioned*.

**adjust:** *tCa: the tCa was corrected by the formula = measured total calcium (mg/dl) + 0.8 × [4.0 – serum albumin (g/dl)]*.

### Variable Selection and Nomogram Construction

The following basic information and serological indicators and the two severity score scales from the MIMIC III program were screened out for investigating the risk factors associated with 28-day mortality in patients with AHF: sex, age, race, the SOFA, the GCS, WBC, Hb, PLT, Na, K, Cl, tCa, BE, ${\text{HCO}}_{3}^{-}$, AG, Lac, BUN, Scr, ALB, TBil, ALT, AST, TnT, and CK-MB. The developed nomogram of risk factors associated with 28-day mortality in patients with AHF is based on two aspects. One was based on the statistically significant different results calculated *via* the multivariate Cox proportional hazard regression analysis (*p* < 0.05). The other was based on the variables, which have been demonstrated significantly associated with cardiovascular-related death, regardless of the two-tailed *p*-value.

### Statistical Analysis

The MIMIC III software was applied to identify the patients who met the inclusion criteria in the MIMIC III program. Baseline characteristics among the AHF death group and non-death group were compared using the Pearson's chi-squared test (minimal expected value > 5) and the Fisher's exact test (minimal expected value ≤5). The least absolute shrinkage and selection operator (LASSO) Cox regression algorithm with 10-fold cross-validation was used to select the optimal variables that were most relevant to the 28-day mortality of patients with AHF. A two-tailed *p*-value of < 0.05 was defined as the criterion for variable deletion when performing backward stepwise selection. The LASSO regression and the multivariate Cox proportional hazard regression analysis were performed by using the “rms” package derived from the “R” software (http://www.r-project.org, R Foundation, Vienna, Austria, version 3.5.3). The Harrell concordance indexes (C-index), which are equivalent to the area under the receiver (AUC) operating characteristic (ROC) curve, were calculated for evaluating the discrimination of the model and the calibration curves were performed to assess the accuracy of the nomogram.

## Results

### Demographic Characteristics of Patients With AHF in the Training Cohort

After excluding, there were 1,371 patients with AHF in the MIMIC III program between 2001 and 2012 and 124 patients with AHF from the Department of Cardiology Internal Medicine in the Second Affiliated Hospital of Chongqing Medical University between January 2019 and April 2021 enrolled in this study. In the training cohort, short-term mortality was observed in 16.5% (226/1,371 cases) of patients. The male patients accounted for a relatively higher rate of suffering AHF compared with the female subpopulations. The elderly patients (age > 65 years, 1,031/1,371 cases, 75.3%) and white race (1,019/1,371 cases, 74.3%) played a predominately part of the AHF population. With respect to the electrolyte indicators, approximately 20% of patients with AHF were identified with electrolyte imbalance. The specific clinical features of the patients in the training and validation cohorts are shown in Table 1.

### Clinical Characteristics Between AHF Death Group and Non-death Group

There were significant differences between the AHF death group and non-death group in terms of age (*p* < 0.001), the SOFA score (*p* < 0.001), and the GCS score (*p* < 0.001) compositions, while no significant difference was identified in terms of race and sex. With respect to the serological indicators, there were significantly differences between the AHF death group and non-death group in terms of serum Cl (*p* = 0.033), BE (*p* < 0.001), ${\text{HCO}}_{3}^{-}$ (*p* < 0.001), AG (*p* < 0.001), Lac (*p* < 0.001), BUN (*p* < 0.001), Scr (*p* < 0.001), ALB (*p* = 0.004), ALT (*p* = 0.013), AST (*p* = 0.006), TnT (*p* < 0.001), and CK-MB (*p* < 0.001) compositions (Table 2).

**Table 2:** Clinical characteristics among the AHF death group and non-death group in the training cohort.

**Variables**	**Subgroup**	**No. (%) of patients**		
		**Death group** **(*n =* 226)**	**Non-death group** **(*n =* 1,145)**	^*^ *P*
Sex	Female	89 (39.4)	506 (44.2)	0.182
	Male	137 (60.4)	639 (55.8)	
Age (years)	≤65	24 (10.6)	316 (27.6)	**<0.001**
	>65	202 (89.4)	829 (72.4)	
Race	White	172 (76.1)	847 (74.0)	0.112
	Black	13 (5.8)	115 (10.0)	
	Other	41 (18.1)	183 (16.0)	
SOFA (score)	≥0 and <2	18 (8.0)	287 (25.1)	**<0.001**
	≥3 and ≤8	140 (61.9)	729 (63.7)	
	≥9 and ≤20	68 (30.1)	129 (11.3)	
GCS (score)	≥13 and ≤15	174 (77.0)	1,012 (88.4)	**<0.001**
	≥9 and ≤12	27 (11.9)	79 (6.9)	
	≥4 and ≤8	25 (11.1)	54 (4.7)	
WBC (k/uL)	<4	5 (2.2)	27 (2.3)	0.149
	≥4 and ≤10	83 (36.7)	499 (43.6)	
	>10	138 (61.1)	619 (54.1)	
Hb (g/L)	Normal	58 (25.7)	366 (32.0)	0.061
	Low	168 (74.3)	779 (68.0)	
PLT (k/uL)	<100	18 (8.0)	80 (7.0)	0.550
	≥100 and ≤300	159 (70.3)	846 (74)	
	>300	49 (21.7)	219 (19.0)	
Na (mmol/L)	Normal	169 (74.8)	905 (79.0)	0.155
	Abnormal	57 (25.2)	240 (21.0)	
K (mmol/L)	<3.5	29 (12.8)	128 (11.2)	0.062
	≥3.5 and ≤5.5	175 (77.4)	950 (83.0)	
	>5.5	22 (9.7)	67 (5.8)	
Cl (mmol/L)	<96	35 (15.5)	111 (9.7)	**0.033**
	≥96 and ≤106	126 (55.7)	698 (61.0)	
	>106	65 (28.8)	336 (29.3)	
^adjust^tCa (mg/dl)	Normal/hypercalcemia	99 (43.8)	506 (44.2)	0.573
	Hypocalcemia	127 (56.2)	639 (55.8)	
BE (mmol/L)	<-3	68 (30.1)	191 (16.7)	**<0.001**
	≥-3 and ≤3	124 (54.9)	753 (65.8)	
	>3	34 (15.0)	201 (17.6)	
${\text{HCO}}_{3}^{-}$ (mmol/L)	<22	98 (43.4)	330 (28.8)	**<0.001**
	≥22 and ≤28	88 (38.9)	599 (52.3)	
	>28	40 (17.7)	216 (18.9)	
AG (mmol/L)	≤16	122 (54.0)	845 (73.8)	**<0.001**
	>16	104 (46.0)	300 (26.2)	
Lac (mmol/L)	≤2	133 (58.8)	897 (78.3)	**<0.001**
	>2	93 (41.1)	248 (21.7)	
BUN (mg/dl)	≤21	21 (9.3)	317 (27.7)	**<0.001**
	>21	205 (90.7)	828 (72.3)	
Scr (mg/dl)	Normal	84 (37.2)	600 (52.4)	**<0.001**
	High	142 (62.8)	545 (47.6)	
ALB (g/dl)	<3.5	199 (88.1)	913 (79.7)	**0.004**
	≥3.5	27 (11.9)	232 (20.3)	
Tbil (mg/dl)	≤1.2	166 (73.4)	908 (79.3)	0.051
	>1.2	60 (26.5)	237 (20.7)	
ALT (U/L)	≤40	139 (61.5)	800 (69.9)	**0.013**
	>40	87 (38.5)	345 (30.1)	
AST (U/L)	≤35	95 (42.0)	595 (52.0)	**0.006**
	>35	131 (58.0)	550 (48.0)	
TnT (ng/ml)	≤0.04	37 (16.4)	654 (57.1)	**<0.001**
	>0.04	189 (83.6)	491 (42.9)	
CKMB (ng/ml)	≤5	77 (34.1)	715 (62.4)	**<0.001**
	>5	149 (65.9)	430 (37.6)	

*SOFA, Sequential Organ Failure Assessment; GCS, Glasgow Coma Scale; WBC, white blood cell; Hb, hemoglobin (normal: male > 120 g/l, female > 110 g/l); PLT, platelet; Na, sodium (normal: 135–145 mmol/l); K, potassium; Cl, chlorine; tCa, total calcium (normal: ≥9 and ≤11 mg/dl); ${\text{HCO}}_{3}^{-}$, bicarbonate; BE, base excess; AG, anion gap; Lac, lactic acid; BUN, blood urea nitrogen; Scr, serum creatinine (normal: male: <1.5 mg/dl, female: <1.0 mg/dl); ALB, albumin; Tbil, total bilirubin; ALT, aspartate aminotransferase; AST, alanine aminotransferase; TnT, troponin T; CK-MB, creatine kinase-MB*.

* *Two-tail Pearson's chi-squared test*.

**adjust:** *tCa: the tCa was corrected by the formula = measured total calcium (mg/dl) + 0.8 × [4.0 – serum albumin (g/dl)]*.

*Bold values indicate statistical significance (p < 0.05)*.

### Least Absolute Shrinkage and Selection Operator Regression Analysis

**Figure 2:**
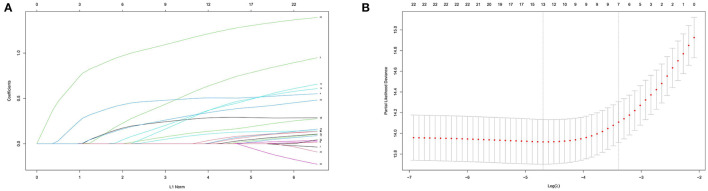
Clinical feature selection using the least absolute shrinkage and selection operator (LASSO) Cox proportional hazard regression analysis with 10-fold cross-validation. **(A)** Tuning parameter selection in the LASSO Cox regression model. **(B)** The LASSO coefficient analysis of the clinical features.

A total of 24 indicators including sex, age, race, the SOFA score, the GCS score, WBC, Hb, PLT, Na, K, Cl, tCa, BE, ${\text{HCO}}_{3}^{-}$, AG, Lac, BUN, Scr, ALB, TBil, ALT, AST, TnT, and CK-MB were initially elected to the LASSO regression algorithm with 10-fold cross-validation (Figure 2). There were 12 predictor variables including age, the SOFA score, the GCS score, Hb, BUN, Lac, ${\text{HCO}}_{3}^{-}$, AG, TBil, ALB, TnT, and CK-MB were selected for inclusion in the multivariate Cox proportional hazard regression analysis model.

### Multivariate Cox Proportional Hazard Regression Analysis

The results indicated that elderly age > 65 years [hazard ratio (HR) = 2.47, 95% CI: 1.61–3.80, *p* < 0.001), the higher SOFA score (≥3 and ≤8: HR = 2.21, 95% CI: 1.33–3.68; ≥9 and ≤20: HR = 3.29, 95% CI: 1.83–5.89, *p* < 0.001), Lac > 2 mmol/l (HR = 1.40, 95% CI: 1.05–1.89, *p* = 0.022), BUN > 21 mg/dl (HR = 1.75, 95% CI: 1.09–2.80, *p* = 0.019), ${\text{HCO}}_{3}^{-}$ > 28 mmol/l (HR = 1.59, 95% CI: 1.09–2.32, *p* = 0.025), ALB < 3.5 g/dl (HR = 2.02, 95% CI: 1.34–3.05, *p* = 0.001), TnT > 0.04 ng/ml (HR = 4.02, 95% CI: 2.74–5.90, *p* < 0.001), and CK-MB > 5 ng/ml (HR = 1.64, 95% CI: 1.21–2.23, *p* = 0.001) were the independent risk factors in predicting the 28-day mortality of intensive care patients with AHF (Table 3).

**Table 3:** The multivariate Cox proportional hazard regression analysis of risk factors associated with 28-day mortality in patients with AHF during the first intensive care unit admission.

**Variables**	**Subgroup**	**Hazard ratio**	***P***
Age (years)	≤65	1	**<0.001**
	>65	2.47 (1.61–3.80)	
SOFA (score)	≥0 and ≤2	1	**<0.001**
	≥3 and ≤8	2.21 (1.33–3.68)	
	≥9 and ≤20	3.29 (1.83–5.89)	
GCS (score)	≥13 and ≤15	1	0.260
	≥9 and ≤12	1.20 (0.79–1.82)	
	≥4 and ≤8	1.42 (0.90–2.26)	
Hb (g/L)	Normal	1	0.281
	Low	1.18 (0.87–1.60)	
${\text{HCO}}_{3}^{-}$ (mmol/L)	<22	0.91 (0.67–1.25)	**0.025**
	≥22 and ≤28	1	
	>28	1.59 (1.09–2.32)	
AG (mmol/L)	≤16	1	0.051
	>16	1.35 (0.99–1.84)	
Lac (mmol/L)	≤2	1	**0.022**
	>2	1.40 (1.05–1.89)	
BUN (mg/dl)	≤21	1	**0.019**
	>21	1.75 (1.09–2.80)	
ALB (g/dl)	<3.5	2.02 (1.34–3.05)	**0.001**
	≥3.5	1	
TBil (mg/dl)	≤1.2	1	0.205
	>1.2	1.22 (0.89–1.67)	
TnT (ng/ml)	≤0.04	1	**<0.001**
	>0.04	4.02 (2.74–5.90)	
CKMB (ng/ml)	≤5	1	**0.001**
	>5	1.64 (1.21–2.23)	

*SOFA, Sequential Organ Failure Assessment; GCS, Glasgow Coma Scale; Hb, hemoglobin (normal: male > 120 g/l, female > 110 g/l); ${\text{HCO}}_{3}^{-}$, bicarbonate; AG, anion gap; Lac, lactic acid; BUN, blood urea nitrogen; ALB, albumin; TBil, total bilirubin; TnT, troponin T; CK-MB, creatine kinase-MB*.

*Bold values indicate statistical significance (p < 0.05)*.

### Nomogram Construction and Validation

**Figure 3:**
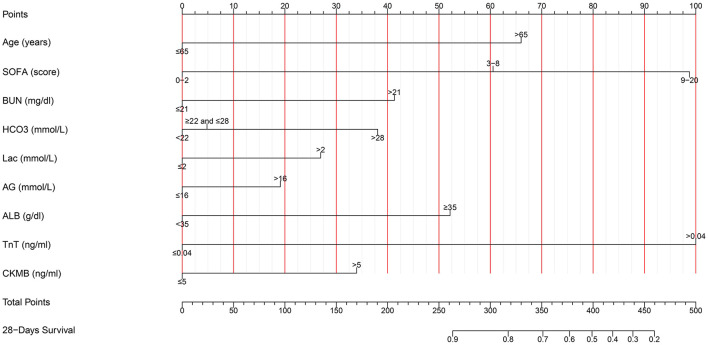
The nomogram used for predicting the short-term mortality in intensive care patients with acute heart failure. SOFA, Sequential Organ Failure Assessment; BUN, blood urea nitrogen; TnT, troponin T; CK-MB, creatine kinase-MB; ${\text{HCO}}_{3}^{-}$, bicarbonate; Lac, lactic acid; AG, anion gap; ALB, albumin.

**Figure 4:**
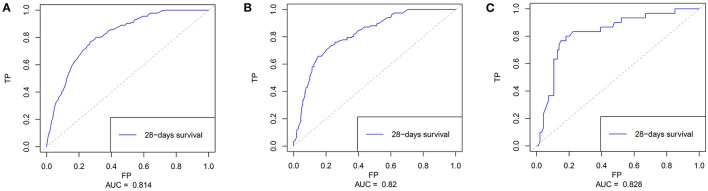
The time-dependent receiver operating characteristics (ROC) curve and area under the ROC curve (AUC). **(A)** The ROC in the training cohort; **(B)** The ROC in the internal validating cohort; and **(C)** The ROC in the external validating cohort.

**Figure 5:**
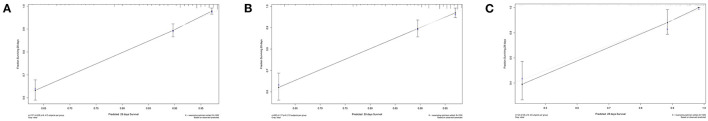
The calibration curves for evaluating the accuracy of the nomogram. **(A)** The calibration curve in the training cohort; **(B)** The calibration curve in the internal validation cohort derived from the 1,000 resampling bootstrap analysis; and **(C)** The calibration curve in the external validation cohort.

Based on the multivariate results, nine variables including age, the SOFA score, and serum levels of BUN, TnT, CK-MB, Lac, ${\text{HCO}}_{3}^{-}$, AG, and ALB were used to construct an intuitive nomogram for predicting the 28-day mortality in intensive care patients with AHF (Figure 3). Every variable was given a score from 0 to 100 and the specific score of each variable is shown in Table 4. Reflecting the discrimination of the nomogram, the C-index, which was in accordance with the AUC of the time-dependent ROC, was above 0.7 and reached 0.814 (95% CI: 0.754–0.882) (Figure 4A). Furthermore, the AUC of the internal validation cohort, which derived from 1,000 resampling bootstrap analysis, also achieved 0.820 (95% CI: 0.721–0.897) (Figure 4B). To evaluate the feasibility of the nomogram in other populations, an external validation cohort from our medical center was further analyzed. The AUC of the external validation cohort also achieved a promising result of 0.828 (95% CI: 0.743–0.917) (Figure 4C). Moreover, to evaluate the utility of the nomogram, three calibration curves of 28-day mortality risk in patients with AHF were displayed. The curves suggested a favorable agreement in the training cohort (Figure 5A) and internal cohort (Figure 5B) and an external cohort (Figure 5C), respectively.

**Table 4:** The specific value of clinicopathological factors in the nomogram of the training cohort.

	**Characteristics**	**Score**
**SOFA**		
	≥0 and <2	0
	≥3 and ≤8	61
	≥9 and ≤20	99
**BUN**		
	≤21	0
	>21	41
${\text{HCO}}_{3}^{-}$		
	<22	0
	≥22 and ≤28	5
	>28	38
**TnT**		
	≤0.04	0
	>0.04	100
**CKMB**		
	≤5	0
	>5	34
**Lac**		
	≤2	0
	>2	27
**AG**		
	≤16	0
>16	19	
**Age**		
	≤65	0
	>65	66
**ALB**		
	<3.5	0
	≥3.5	52
**Total point for 28-day survival**		
	0.2	460
	0.3	439
	0.4	419
	0.5	399
	0.6	377
	0.7	351
	0.8	317
	0.9	263

*SOFA, Sequential Organ Failure Assessment; BUN, blood urea nitrogen;* ${\text{HCO}}_{3}^{-}$, *bicarbonate; TnT, troponin T; CK-MB, creatine kinase-MB; AG, anion gap; ALB, albumin*.

## Discussion

Acute heart failure is a severe clinical syndrome characterized as rapid onset or worsening of symptoms of chronic HF, which is the leading cause of unplanned hospital admission and even readmission in patients aged > 65 years (2, 3, 21–23). Compared with the promising improvements in the treatment of chronic HF, AHF is still associated with poor prognosis regardless of therapeutic advances (13, 24). According to the recent comprehensive review reports (2, 3), the overall in-hospital mortality of AHF ranged from 4 to 7%, whereas the highest mortality rate (reaching 17.8%) was identified in patients with ICU (25). Besides, the risk of mortality rises after hospital discharge with ~10% mortality at 30-days and 22–27% mortality at 1 year (2). Thus, there is an urgent need for early clinical assessment, risk stratification, and increased individualization and continuation of treatment after hospital discharge to improve long-term outcomes in patients with AHF.

In this study, we ultimately included 1,371 patients with the diagnosis of AHF at initial admission to ICU from the MIMIC III program. The short-term (28-day) mortality rate was 16.5% (226/1,371 cases), which was higher than the result of Miró et al. (11) from a prospective cohort study (10.3%), but slightly lower than Follath et al. (25) results from the Acute Heart Failure Global Registry of Standard Treatment (ALARM-HF) trial (17.8%). Among the AHF death group and non-death group, there were significant differences between the groups in terms of age (*p* < 0.001), the SOFA score (*p* < 0.001), and the GCS score (*p* < 0.001) compositions, while no significant difference was identified in terms of race and sex. Interestingly, we did not determine the significant difference in sex or race composition of the two groups. However, in one large-scale population-based study with a 1 year follow-up, Sun et al. (26) demonstrated the different patterns of ethnicity in patient outcomes with AHF. They concluded a lower risk of 1 year mortality after AHF hospitalization among South Asians compared to Chinese and the general population and similar benefits of medical therapy in all the three groups. On the other hand, with respect to the serological examination, our results partially confirmed the findings derived from the previous studies and took it a step further. Notably, significantly differences were identified in serological indicators in terms of serum Cl (*p* = 0.033), BE (*p* < 0.001), ${\text{HCO}}_{3}^{-}$ (*p* < 0.001), AG (*p* < 0.001), Lac (*p* < 0.001), BUN (*p* < 0.001), Scr (*p* < 0.001), ALB (*p* = 0.004), ALT (*p* = 0.013), AST (*p* = 0.006), TnT (*p* < 0.001), and CK-MB (*p* < 0.001) among AHF death group and non-death group in this study.

Furthermore, based on the multivariate Cox proportional hazard regression analysis results, we confirmed eight variables that presented a significant association with the risk of short-term in-hospital mortality in patients with AHF. The age, the SOFA score, and TnT level were the three leading predictors of the short-term mortality of patients with AHF in ICU. Especially, the SOFA score was a composite and utility clinical–biological score, which could help clinicians to access the potential risk of patients. Most recently, Elias et al. (27) determined the feasibility of the SOFA score (AUC: 0.765) in predicting the short-term mortality in patients with AHF. Our results also yielded that the SOFA score could be used as a complementary risk score to early identify high-risk patients who need strict management. Although the GCS score and serum levels of AG and Scr showed statistically significant differences during the univariate analysis, these differences disappeared after adjustment for the other factors. Nonetheless, there was a tendency toward an increased risk of mortality in patients with high levels of AG [odds ratio (OR) = 1.42, *p* = 0.092]. Moreover, according to the evidence derived from the Epidemiology of Acute Heart Failure in Emergency departments (EAHFE) registry (17), the high-sensitive (hs)-TnT was recently confirmed to be an optimal biomarker in predicting the 30-days all-come mortality in patients with AHF (with the best cutoff point of 35 ng/l). A similar result was also displayed in one recent multicenter-based study, while the cutoff point was hs-TnT ≥ 43 ng/l (28). Although the indicator in this study was not hs-TnT but TnT (>0.04 ng/ml, HR = 4.02), it supported the promising predicting value of this serum biomarker.

One recent study concluded that it was pivotal to correct the calcium level in patients with AHF, which could help to reduce the misdiagnosis of hypocalcemia (29). In this study, concerning the impact of albumin on the calcium level, especially in patients with hypoalbuminemia, the calcium level reported was corrected for albumin [measured total calcium (mg/dl) + 0.8 × 4.0 – serum albumin (g/dl)]. After adjusting, a significant decrease in proportion of patients with hypocalcemia was observed. A similar result was also determined in our medical center (hypocalcemia rate decreased from 50.8 to 33.9%). Previous reports, especially case reports, mentioned the association between hypocalcemia and hypercalcemia and AHF (29–32). Thus, future studies are needed to evaluate its predicting value in mortality of AHF and the potential mechanisms behind it. Besides, we also confirmed the correlation between albumin level and survival of patients with HF. There were several potential explanations for the relationship between hypoalbuminemia and the survival of AHF. Hypoalbuminemia was frequently occurred in advanced age, malnutrition, and inflammation, which were known to predict a worse prognosis of AHF (33, 34). Moreover, decreased colloid osmotic pressure caused by hypoalbuminemia could lead to the development of pulmonary edema and exacerbation of AHF. Furthermore, patients with hypoalbuminemia usually present a worse prognosis in several multimorbidity conditions such as late-stage renal disease, infection, and cancer, which were highly prevalent in elderly patients with AHF and could contribute to their increased mortality risk. As for BUN, compelling evidence has demonstrated a positive correlation between BUN and increased mortality of AHF (2, 3). The high level of BUN predicted a worse renal function, which could further impair the circulation and metabolism of the body and aggravate the symptoms of AHF.

Nowadays, individual biomarkers can be utilized to predict clinical outcomes in patients with AHF, including but not limited to the risk of mortality and readmission (5, 9–11, 34–36). In one earlier study, based on classification and regression tree analysis, Fonarow et al. (37) applied a model within only three factors to the clinical practice including BUN (>43 mg/dl), low admission systolic blood pressure (<115 mm Hg), and high levels of Scr (>2.75 mg/dl). With more clinical variables [using the American Heart Association Get With the Guidelines-Heart Failure (GWTG-HF) program data] involvement, they constructed another new model within seven indicators such as age, systolic blood pressure, BUN, heart rate, Na, COPD, and non-black race for predicting in-hospital mortality (7). Also, in one Spanish trial (11), the Multiple Estimation of Risk Based on the Emergency Department Spanish Score in Patients with AHF (MEESSI-AHF) scores included 13 independent risk factors to estimate the 30-day mortality in patients with AHF and achieved a C-index of 0.836. However, serological indicators combined with severity scores could be sufficient to predict the short-term risk of in-hospital mortality was rarely explored. Additionally, in China, contemporary data on the epidemiology of HF including AHF in China are scarce with only a few studies that could be reached (38, 39).

Additionally, to vividly display the results from multivariate analysis, we further constructed a prediction model for clinical use. Some prior studies mentioned above have constructed risk score models for predicting the in-hospital mortality or postdischarge prognosis in patients hospitalized with AHF (11, 34, 37). However, few researchers, to the best of our knowledge, have ever attempted to establish a nomogram that was frequently used to predict metastasis and survival in the oncology field (40, 41) to visualize the prognostic factors with different scores. For this reason, we filled this gap and developed a nomogram with nine predictors of involvement for predicting the short-term in-hospital mortality in patients with AHF. Optimistically, the C-index of the model, which was in accordance with the AUC, was above 0.70 and reached 0.795 (95% CI: 0.711–0.898). It indicated a favorable discrimination ability of our model to identify patients with AHF at high risk. Besides, an internal cohort *via* 1,000 resampling bootstrap and an external validation cohort from our medical center also proved the utility of the nomogram. Our risk model had a higher C-index than models established by Elias et al. (27) (C-index: 0.765) and Peterson et al. (7) (C-index: 0.750). Although the AUC of the model made by Kinugasa et al. (34) was 0.860, the sample size was only 349 cases and all of them were aged over 65 years. Thus, we suggest that this novel nomogram could help clinicians to identify the patients who are at high risk for death once they were admitted to the ICU and CCU.

Nevertheless, this study has some limitations, which need to be mentioned. First, the data of brain natriuretic peptide (BNP), N-terminal pro-BNP (NT-proBNP) (42) were missing in the MIMIC III program, which could be added combined with other novel biomarkers including but not limited to soluble ST2 (sST2), growth-differentiation factor-15 (GDF-15), cystatin C, galectin-3, serum uric acid, microRNAs, and low serum chloride in the further update (9, 43). Second, the distinguishment of HF with reduced ejection fraction (HFrEF) or HF with preserved ejection fraction (HFpEF) left ventricular ejection fraction (LVEF) was not recorded in the MIMIC III program. Thus, this model could be applied to both the two conditions that need further evaluation. Third, although the training data was derived from a multipopulational program, the retrospective nature of this study introduced the possibility of inherent observational and selection bias. Moreover, the nomogram was established by dichotomizing continuous variables. Thus, the individualized score should be cautious to interpret during clinical practice. Last, while an external cohort from a single medical center has confirmed the utility of the nomogram, the cases of the validation cohort were relatively small. Therefore, the nomogram established needs further robust prospective validation with larger sample size.

## Conclusion

This study indicates that elderly age (>65 years), the high SOFA score (>3), ${\text{HCO}}_{3}^{-}$ > 28 mmol/l, Lac (>2 mmol/l), BUN (>21 mg/dl), albumin (<3.5 g/dl), TnT (>0.04 ng/ml), and CK-MB (>5 ng/ml) are the independent risk factors in 28-day mortality of patients with AHF. Among these indicators, age > 65 years (HR = 2.47), the SOFA score ≥ 9 (HR = 3.29), and TnT (HR = 4.02) are the leading three predictors of 28-day mortality of patients with AHF. Additionally, we develop and further validate a nomogram for individualized predicting the short-term mortality once patients with AHF are admitted to the ICU. Patients at high risk of mortality are supposed to assign a higher level of active monitoring and earlier and a more intensive treatment. Meanwhile, patients estimated to have a relatively good prognosis may be suitable candidates for routine treatment, although individual factors and preferences of the patient would still require careful consideration.

## Data Availability Statement

The raw data supporting the conclusions of this article will be made available by the authors, without undue reservation.

## Author Contributions

YM, XW, QW, HW, and SL organized the database. YM, XW, and JY performed the statistical analysis. All authors contributed to the conception and design of the study, wrote the first draft of the manuscript, wrote sections of the manuscript, contributed to manuscript revision, read, and approved the final version of the manuscript.

## Conflict of Interest

The authors declare that the research was conducted in the absence of any commercial or financial relationships that could be construed as a potential conflict of interest.

## Publisher's Note

All claims expressed in this article are solely those of the authors and do not necessarily represent those of their affiliated organizations, or those of the publisher, the editors and the reviewers. Any product that may be evaluated in this article, or claim that may be made by its manufacturer, is not guaranteed or endorsed by the publisher.

## Supplementary Material

Supplementary Figure 1 The proportional hazards assumption was assessed by plotting Schoenfeld residuals vs. time and examining their correlation.
